# Cytokine Concentrations in Plasma from Children with Severe and Non-Severe Community Acquired Pneumonia

**DOI:** 10.1371/journal.pone.0138978

**Published:** 2015-09-25

**Authors:** Johanne Haugen, Ram K. Chandyo, Karl A. Brokstad, Maria Mathisen, Manjeswori Ulak, Sudha Basnet, Palle Valentiner-Branth, Tor A. Strand

**Affiliations:** 1 Department of Medical Microbiology, Innlandet Hospital Trust, Lillehammer, Norway; 2 Broegelmann Research Laboratory, Dept. of Clinical Sciences, University of Bergen, Bergen, Norway; 3 Centre for International Health, University of Bergen, Bergen, Norway; 4 University hospital of Northern Norway, Tromsø, Norway; 5 Department of Child Health, Institute of Medicine, Tribhuvan University, Kathmandu, Nepal; 6 Department of Infectious Disease Epidemiology, Statens Serum Institut, Copenhagen, Denmark; University of Tennessee Health Science Center, UNITED STATES

## Abstract

**Background:**

Children in low and middle-income countries have a high burden of pneumonia. Measuring the cytokine responses may be useful to identify novel markers for diagnosing, monitoring, and treating pneumonia.

**Objective:**

To describe and compare a wide range of inflammatory mediators in plasma from children with WHO-defined severe and non-severe community acquired pneumonia (CAP), and explore to what extent certain mediators are associated with severity and viral detection.

**Methods:**

We collected blood samples from 430 children with severe (n = 43) and non-severe (n = 387) CAP. Plasma from these children were analysed for 27 different cytokines, and we measured the association with age, disease severity and viral detection.

**Results:**

There were generally higher plasma concentrations of several cytokines with both pro-inflammatory and anti-inflammatory effects among children with severe CAP than in children with non-severe CAP. We found significantly higher concentrations of interleukin (IL)-1, IL-4, IL-6, IL-8, IL-9, IL-15, eotaxin, basic fibroblast growth factor (b-FGF), granulocyte colony-stimulating factor (G-CSF), granulocyte-macrophage colony-stimulating factor (GM-CSF), and tumor necrosis factor-alpha (TNF-α) in the group of severe CAP. Most of these associations persisted when adjusting for age in linear regression analyses. The cytokine response was strongly associated with age but to a lesser extent with viral etiology.

**Conclusion:**

The plasma concentrations of several cytokines, both with pro-inflammatory and anti-inflammatory effects, were higher among children with severe illness. In particular G-CSF and IL-6 reflected severity and might provide complementary information on the severity of the infection.

**Trial registration:**

ClinicalTrials.gov NCT00148733

## Introduction

The burden of community-acquired pneumonia (CAP) in children is substantial in low- and middle-income countries. Despite a 25% reduction in total cases progressing to severe episodes in the past decade, childhood pneumonia is still one of the leading causes of death. Pneumonia and diarrhoea combined claimed the lives of more than 1.7 million under-five children in 2012 [1, 2]. An immature immune system makes young children particularly susceptible to infections [3, 4]. Because of a wide range of etiological agents, difficulties of obtaining representative samples, and knowledge gaps in understanding why some cases develop into severe or very severe cases, there are challenges to both diagnosing and managing childhood pneumonia.

The cytokine responses in CAP have been explored mostly in adults and only a few studies have examined the cytokine response in young children with respiratory infections. One study in children with CAP found that systemic IL-6 was elevated and associated with markers of severity measured by white blood cell-band- forms, elevated procalcitonin and unequivocal consolidation [5]. One study on cytokine profile and pandemic influenza H1N1 2009 virus infection in paediatric patients with pneumonia demonstrated that the concentration of interferon gamma inducible potein-10 (IP-10) and IL-6 concentrations were proportional to the severity of the infection defined by lymphopenia and hypoxia [6]. Another study found higher concentrations of IL-10 and IL-5 in H1N1-infected patients with pneumonia compared to H1N1-infected patients without pneumonia [7].

Previous studies of cytokine profiles in pneumonia have mostly been based on selected hospital populations in developed countries and have analysed only a few cytokines. As most paediatric pneumonia cases and pneumonia related deaths are in the developing world, we wanted to describe the cytokine concentrations in WHO-defined severe and non-severe pneumonia in Nepalese children aged 2–35 months. In order to make a comprehensive description of the inflammatory response we analyzed the plasma concentrations of 27 cytokines. We also explored the association of cytokine concentrations and certain background variables and viral detection.

## Materials and Methods

### Ethical clearance

Ethical clearance was given by the Institute of Medicine, Tribhuvan University in Kathmandu, Nepal, and the Regional Committee for Medical and Health Research Ethics in the western part of Norway, REC West. The implementation of all aspects of the project was in agreement with the International Ethical Guidelines for Research Involving Human Subjects as stated in the latest version of the Helsinki Declaration. Informed written consent was obtained from one of the parents. A witnessed verbal informed consent was obtained from those who were illiterate. The ethic committees approved this procedure.

### Study population and study design

This study was planned as secondary analyses of data collected in a previously completed randomized double blind, placebo-controlled trial (RCT) in Bhaktapur, Nepal (NCT00148733) [8]. In this RCT, 2628 children were randomized to receive zinc (10 mg /d for infants and 20 mg /d for children 12 months or older) or placebo for 14 days. The primary objective of the RCT was to measure the clinical effect of oral zinc supplementation in children with severe and non-severe pneumonia as defined by the WHO [9]. Out of 2628 patients, 430 were randomly selected to have blood samples, and the description of baseline concentrations of inflammatory mediators was a predefined secondary outcome. The number 430 was based upon power calculations for estimating the effect of oral zinc administration on plasma zinc levels. The target population of this study was children aged 2–35 months in the study area in Bhaktapur district in the Kathmandu valley, Nepal. The study population consisted of children with pneumonia, attending the study clinic established at Siddhi Memorial Hospital because of cough and / or difficulty breathing. Non-severe pneumonia was diagnosed by the criteria cough or breathing difficulties and age-adjusted tachypnea (for children aged 2–11 months defined as ≥ 50 breaths/min and for children aged ≥ 12 months defined as ≥ 40 breaths/min). Presence of chest indrawings or other danger signs (as unable to feed or drink, vomiting everything, convulsions, lethargic or unconscious) was used as a marker of severe pneumonia. Children with wheezing were given two doses of nebulized salbutamol and reassessed after 30 min whether he or she still fulfilled the inclusion criteria. Nonconsent, not planning to live in the area for the next 6 months, requiring care for very severe disease (ie, any general danger sign), severe malnutrition (defined as being < 70% of the median weight for height according to National Centre for Health Statistics standards), presence of congenital heart disease, documented tuberculosis, documentation of any oral antibiotic treatment in the past 48 h, cough for >14 days, severe anaemia (defined as haemoglobin < 7 g/dL), or dysentery were exclusion criteria.

### Data collection

A study physician at the study clinic screened children eligible for inclusion. The history of the child’s illness and a physical examination was carried out using a standardized form. Height and weight were measured and anthropometric measures expressed as Z-scores were generated by using WHO Child Growth Standards [10]. Respiratory rate was counted according to WHO guidelines [9], and oxygen saturation was measured by a pulse-oximeter with a paediatric sensor. Capillary or venous blood (3–4.5 mL) was obtained for measurement of haemoglobin and CRP and plasma was frozen to later analysis of inflammatory markers. Nasopharyngeal aspirates were taken. Treatment was given according to the WHO case management guidelines for pneumonia [9]. Children with non-severe pneumonia were given per oral co-trimethoxazole /kg bodyweight twice daily for 5 days and trained fieldworkers under supervision from study physicians visited the families daily for examination and monitoring of the child. Illness characteristics as respiratory rate, axillary temperature, development of LCI or any danger sigs were recorded. If fast breathing persisted after 72 hours or the child got worse he or she was referred to the clinic and reassessed by a study physician. The treatment was changed to per oral amoxicillin if the child still fulfilled the criteria of non-severe pneumonia, alternatively the child was hospitalized if there was development of severe pneumonia or other conditions requiring hospitalization. The parents were also advised to bring their child to the clinic whenever needed. Hospitalized children were given treatment with parenteral benzyl penicillin for 3 days and other supportive treatment as required. If there was no improvement within 48 hours the treatment was changed to chloramphenicol. Study physicians examined the hospitalized children with 12 hours intervals, and illness characteristics as oxygen saturation, respiratory rate, auscultatory findings and presence of LCI and other danger signs were recorded. Recovery of non-severe pneumonia was defined as the first of 2 consecutive days with normal respiratory rate, and recovery from severe pneumonia was defined as the beginning of the first 24-hour period without LCI, without nasal flaring and without grunting. The children recovering from severe pneumonia were discharged and given oral antibiotic treatment for a course of 10 days, and daily monitored at home by a fieldworker until recovering from non-severe pneumonia. The plasma specimens were transported on dry ice and then kept frozen at -70 degrees Celsius until later analysis. Nasopharyngeal aspirates were analysed at the laboratory of Tribhuvan University Hospital, Kathmandu, using a multiplex polymerase chain reaction (PCR) for seven common respiratory viruses (Influenza A, Influenza B, respiratory syncytial virus (RSV), human metapneumovirus (hMPV), parainfluenza virus (PIV) type 1, 2 and 3) [11]. Finally, completed forms with family history and all clinical recordings and results were double entered into a database.

### Cytokine analyses

Cytokine analyses in plasma were performed at Broegelmanns Research Laboratory, University of Bergen, using magnetic bead-based multiplex immunoassay for Luminex (LX1000). The kits used were the premade “Bio-Plex Pro™ Human Cytokine Standard 27-Plex, Group I-kit” from BioRad, catalogue number M50-0KCAF0Y. Data from the reactions were acquired using the Luminex reader, while a digital processor managed the data output and the Luminex software returned data as median fluorescence intensity (MFI) and concentration (pg/ml). The following cytokines were implemented in the Bio-Plex Pro™ Human Cytokine Standard 27-Plex, Group I-kit: IL-1β, IL-1ra, IL-2, IL-4, IL-5, IL-6, IL-7, IL-8, IL-9, IL-10, IL-12 (p70), IL-13, IL-15, IL-17A, bFGF, eotaxin, G-CSF, GM-CSF, Interferon-gamma (IFN-γ), IP-10, MCP-1 MIP-1α, MIP-1β, RANTES (regulated on activation, normal T-cell expressed and secreted), TNF-α, platelet derived growth factor subunit B (PDGF-BB) and vascular endothelial growth factor (VEGF). For results under the limits of detection (LOD), the 50%-value of the lowest standard for each of the cytokines was used. Analyses were done according to the Bio-Plex Pro™Human Cytokine, Chemokine, and Growth Factor Assays Instruction Manual.

### Variables

The outcome variables in this study were cytokine concentrations and differences at baseline in the two groups of non-severe and severe pneumonia as defined by the WHO [9]. We also estimated differences in cytokine concentrations in sub-groups based on clinical variables assumed to be associated to severity of pneumonia (CRP, SpO_2_, presence of crepitations and wheezing) and relevant background variables with potential impact on cytokine profiles (age, z-scores, breastfeeding status and exposure to indoor smoking). The cut-off for CRP was based on the practice on the local hospital and the cut-off for SpO_2_ was based on the definition of hypoxia from WHO [12].

### Statistics

Statistical analyses were undertaken using STATA, version 13. Clinical data was expressed as means ± SD or medians with interquartile ranges (IQR) and Fisher´s exact test and Mann-Whitney U-test were used to calculate baseline differences. Medians with IQR were used to express the immune mediator levels. Comparison between the groups of severe and non-severe pneumonia was performed using the non-parametric Mann-Whitney U-test. We also compared different sub-populations based on CRP as a dichotomous variable with a cut-off of 40, SpO_2_ as a dichotomous variable with a cut-off of 90%, presence of crepitations and wheezing, breastfeeding status and nutritional status and exposure to indoor smoking. Multiple linear regressions were used to measure the associations between cytokine concentrations adjusting of other relevant variables. A p-value of < 0.05 was considered statistically significant. In the linear regression models we used the natural log-transformed cytokine concentrations. We also depicted the relationship between cytokine concentrations and age using the “lpoly” plot- command in STATA.

## Results

### Baseline characteristics

The mean (± SD) age of the children was 13.5 (± 8.6), and the children in the group with severe pneumonia were significant younger than the children in the group with non-severe pneumonia (p < 0.001). Of the children included 53% were boys and 94.9% were still breastfed, 66 (15.3%) were stunted (< -2 Z scores for height/length for age) and 19 (4.4%) were wasted (< -2 Z scores for weight for height/length), 387 (90%) had non-severe and 43 (10%) had severe pneumonia. Of the children with severe pneumonia, 42 presented with lower chest indrawings and one fulfilled the criteria because of other general danger signs. The median (IQR) peripheral capillary oxygen saturation (SpO_2_) was 93.5% (91.5–96.5%). Of the children, 12 (2.8%) had hypoxia as defined by the WHO [12] with a SpO_2_ < 90%, the median (IQR) CRP was 12 (0–28), 62 (14.4%) had CRP ≥ 40 mg/L and 16 (3.7%) had CRP ≥ 80 mg/L. The median (IQR) time till recovery for the children in the group with non-severe pneumonia was 3 (2–5) days and 6 (4–8) days for those with severe pneumonia. At least one respiratory virus was detected in NPA from 169 (44%) children, of which 20 (5.2%) were PIV 1, 5 (1.3%) were PIV 2, 32 (8.3%) were PIV 3, 24 (6.3%) were influenza A, 16 (4.2%) were influenza B, 60 (15.6%) were RSV and 16 (4.2%) were hMPV. Two respiratory viruses were detected in NPA from 4 children (Table 1).

**Table 1:** Demographic data, clinical characteristics and viral isolation among children with pneumonia in Bhaktapur, Nepal.

Child characteristics	Designation	Total		Non-	severe	Severe		p
		n	Value	n	Value	n	Value	
Age of child in months	mean ±SD	430	13.5 ± 8.6	387	14.1 ± 8.5	43	7.7 ± 7.4	**<0.001**
Boys	n (%)	430	229 (53.0)	387	205 (53.0)	43	26 (60.5)	0.75
Breastfed yes	n (%)	430	408 (94.9)	387	367 (94.8)	43	41 (95.3)	1.0
Axillary temperature in°C	mean ±SD	430	37.4 ± 0.9	387	37.3 ± 0.9	43	37.3 ± 0.7	0.617
Duration of cough in days	mean ±SD	430	3.1 ± 1.9	387	3.2 ± 2.0	43	3.1 ± 1.8	0.981
Duration of difficulty breathing in days	mean ±SD	430	1.73 ± 1.8	387	1.7 ± 1.9	43	1.8 ± 1.5	0.379
Duration of fever in days	median (IQR)	430	2 (1–3)	387	2 (1–3)	43	2 (1–3)	0.285
Respiratory rate pr/min	mean ±SD	430	54.2 ± 7.9	387	53 ± 6.7	43	66.0 ± 9.2	**<0.001**
Presence of lower chest indrawing (LCI)	n (%)	430	42 (9.8)	387	0 (0.0)	43	42 (97.7)	**<0.001**
SpO_2_	median (IQR)	430	93.5 (91.5–96.5)	387	93.5 (91.5–96.5)	43	91.5 (90.5–95.0)	**0.005**
Hypoxia (SpO_2_ <90%)	n (%)	430	12 (2.8)	387	3 (0.8)	43	9 (20.9)	**<0.001**
CRP	median (IQR)	430	12 (0–28)	387	12 (0–26)	43	25 (0–50)	**0.015**
CRP mg/L >80	n (%)	430	16 (3.7)	387	12 (3.1)	43	4 (9.3)	0.065
CRPmg/L >40	n (%)	430	62 (14.4)	387	48 (12.4)	43	14 (32.6)	**0.001**
Family ownership of land	n (%)	429	205 (47.8)	387	185 (47.8)	43	20 (46.5)	1.0
Living in nuclear family (*)	n (%)	429	212 (49.4)	387	188 (48.6)	43	24 (55.8)	0.397
Number of family members	mean ±SD	429	6.1 ± 3.3	387	6.2 ± 3.3	43	5.5 ± 3.0	0.136
Indoor smoking	n (%)	428	248 (57.9)	386	248 (64.2)	42	22 (52.4)	0.511
Z-score weights for age	mean ±SD	430	-0.8 ± 1.1	387	-0.8 ± 1.1	43	-0.8 ± 1.3	0.968
Z-score length/height for age	mean ±SD	430	-1.1 ± 1.2	387	-1.1 ± 1.2	43	-0.7 ± 1.5	0.077
Z-score weight for length/height	mean ±SD	429	-0.3 ± 1.0	386	-0.3 ± 1.0	43	-0.5 ± 1.1	0.061
< -2 Z-score length/height for age (stunted)	n (%)	430	66 (15.3)	387	85 (22.0)	43	10 (23.3)	0.847
< -2 Z-score weight for length/height (wasted)	n (%)	429	19 (4.4)	386	18 (4.7)	43	1 (2.3)	0.708
Positive for any virus in nasopharyngeal aspirate	n (%)	384	169 (44)	343	147 (42.9)	42	22 (52.4)	0.253
Positive for PIV 1	n (%)	384	20 (5.2)	343	19 (5.5)	42	1 (2.4)	0.71
Positive for PIV 2	n (%)	384	5 (1.3)	343	4 (1.2)	42	1 (2.4)	0.441
Positive for PIV 3	n (%)	384	32 (8.3)	343	31 (9.0)	42	1 (2.4)	0.231
Positive for Influenza A	n (%)	384	24 (6.3)	343	22 (6.4)	42	2 (4.8)	1.0
Positive for Influenza B	n (%)	384	16 (4.2)	343	15 (4.4)	42	1 (2.4)	1.0
Positive for RSV	n (%)	384	60 (15.6)	343	44 (12.8)	42	16 (38.1)	**<0.001**
Positive for hMPV	n (%)	384	16 (4.2)	343	16 (4.7)	42	0 (0.0)	0.236
Positive for two viruses	n (%)	384	4 (1)	343	4 (1.2)	42	0 (0.0)	1.0
Baseline plasma Zinc in μg/dL	mean ±SD	426	58.2 ± 17.3	383	58.1 ± 15.1	43	58.7 ± 31.2	0.235
Time till recovery (days)	median (IQR)	430	3 (2–5)	387	3 (2–5)	43	6 (4–8)	**<0.001**

For p-values Fisher´s exact test was used for dichotomous variables and Mann-Whithey-U-test for continuous variables.

* Nuclear family = children living together with their parents.

### Plasma-cytokine concentrations from children with non-severe and severe pneumonia

We detected all 27 cytokines in the plasma samples and the results are shown in Table 2, together with the limits of detection (LOD) and % < LOD for each cytokine. For RANTES the percentage of samples below the lower limit of detection (LOD) were 94.8%, and we chose therefore not to present the association between RANTES and the baseline variables (Table 2).

**Table 2:** Cytokine concentrations among children with severe and non-severe pneumonia in Bhaktapur, Nepal.

Cytokine	LOD	% <LOD	Severe	pneumonia		Non-	severe	pneumonia	p
	(pg/ml)		n	Median	IQR	n	Median	IQR	
IL-1β	0.6	4.2	43	2	1.2–3.7	383	1.4	1.0–2.3	**0.007**
IL-1ra	5.5	0.4	43	353.4	183.1–962	382	282.8	155.3–566.2	0.102
IL-2	1.6	1	43	20.8	13.3–33.1	382	21.8	13.08–35.0	0.696
IL-4	0.7	1.1	43	2.5	1.9–3.5	383	2	1.3–2.9	**0.008**
IL-5	0.6	3.8	43	2.9	1.2–8.9	383	2	0.6–5.5	0.052
IL-6	2.6	0.3	43	58.4	36.5–90.8	382	39.6	23–76.2	**0.014**
IL-7	1.1	1.4	43	9.3	5–14.6	381	6.7	3.7–11.2	0.087
IL-8	1	0.3	43	35.9	21.9–64.4	382	23.4	17.5–34.2	**<0.001**
IL-9	2.5	0.4	43	45.4	31.3–142.9	382	34.5	25.3–52.1	**0.006**
IL-10	0.3	0	43	28.2	16.9–57.4	383	26.3	15.9–46.5	0.478
IL-12p70	3.5	0.9	43	27.6	20.1.- 56.0	383	31	19.2–52.7	0.91
IL-13	0.7	0.4	43	7.3	4.9–12.1	383	6	3.5–9.7	0.067
IL-15	2.4	30	43	12.8	5.8–29.4	383	5.3	0.9–15.7	**0.001**
IL-17a	3.3	0.4	43	42.4	31.6–58.8	381	38.2	25.1–52.0	0.099
eotaxin	1.9	2.2	43	63.8	43.9–91.4	382	52.15	32.2–80.9	**0.038**
b-FGF	2.5	0.3	43	121.6	94.5–153.1	383	98.4	77.5–128.6	**0.002**
G-CSF	1.7	0.8	43	113.5	54.6–315.2	383	69.1	38.5–135.1	**<0.001**
GM-CSF	2.2	2.6	43	677.6	429.8–923.7	383	465.2	247.1–731.7	**<0.001**
IFN-γ	6.4	0.3	43	96.2	66.7–262.6	383	83.2	51.5–157.5	0.059
IP-10	6.1	0.3	43	1577.4	66.7–262.6	382	1679.2	889.4–3040.8	0.938
MCP-1	1.1	0.3	43	209.8	1186.6–2570.8	383	179	121.8–274.8	0.431
MIP-1α	1.6	0.4	43	4.7	121.1–347.9	382	4.4	3.8–5.7	0.203
MIP-1β	2.4	0.4	43	107.8	3.9–6.3	382	97.05	77.5–137.6	0.769
PDGF-BB	2.9	0.3	43	1277.8	76.1–128.6	382	1348.4	77.5–2348.1	0.309
RANTES	1.8	94.8	43	<LOD		384	<LOD		
TNF-α	6	0.5	43	24.1	13.6–51.9	382	17	12–26.6	**0.009**
VEGF	3.1	0.5	43	101.5	63–135.8	382	87	59.1–138.1	0.563

p-values are based on Mann–Whitney U- test. IL-1b = interleukin-1 beta; IL-1ra = interleukin-1 receptor antagonist; IL-2 = interleukin-2; IL-4 = interleukin-4; IL-5 = interleukin-5; IL-6 = interleukin-6; IL-7 = interleukin-7; IL-8 = interleukin-8; IL-9 = interleukin-9; IL-10 = inter- leukin-10; IL-12 = interleukin-12; IL-13 = interleukin-13; IL-17 = interleukin-17; FGF = fibroblast growth factor; IFN-γ = interferon gamma; IP-10 = interferon gamma-induced protein 10; MCP-1 = monocyte chemotactic protein-1; MIP-1b = macrophage inflammatory protein 1beta; PDGF-BB = platelet-derived growth factor-BB; RANTES = regulated on activation, normal T-cell expressed and secreted; TNF = tumor necrosis factor; VEGF = vascular endothelial growth factor, IQR = inter- quartile range, n = number of observations, LOD = lower limit of detection. (Boxplots are shown as supplemental material, S1 Fig).

Children with severe pneumonia had significantly higher concentrations of IL-1 (p = 0.007), IL-4 (p = 0.008), IL-6 (p = 0.014), IL-8 (p <0.001), IL-9 (p = 0.006), IL-15 (p = 0.001), eotaxin (p = 0.038), b-FGF (p = 0.002), G-CSF (p < 0.001), GM-CSF (p < 0.001), and TNF- (p = 0.009) than children with non-severe pneumonia (Table 2).

Most of the estimates for the associations between severity and these cytokines were relatively unchanged when adjusting for age in the linear regression models. However the associations between severity and the concentrations of IL-1, IL-5, IL-6, eotaxin, GM-CSF was substantially attenuated (Table A in S1 File).

### Variables associated to cytokine concentration at baseline

Children with elevated CRP ≥ 40 mg/L had significantly higher IL-6 (p < 0.001) and G-CSF (p < 0.001) and significantly lower concentrations of IP-10 (p = 0.007), MCP-1 (p = 0.026) and MIP-1β (p = 0.019) than children with CRP < 40. Except for MIP-1β all these associated were present even after adjusting for age. Children with SpO_2_ < 90% also had significantly higher concentrations of IL-1ra (p = 0.010), IL-2 (p = 0.036), IL-5 (p = 0.009), IL-6 (p = 0.012), IL-7 (p = 0.045), IL-9 (p = 0.019), G-CSF (p = 0.018). Except for MIP-1β all these associations were present even after adjusting for age. Children with SpO_2_ < 90% also had significantly higher concentrations of IL-1ra (p = 0.010), IL-2 (p = 0.036), IL-5 (p = 0.009), IL-6 (p = 0.012), IL-7 (p = 0.045), IL-9 (p = 0.019), G-CSF (p = 0.018) and TNF-a (p = 0.018) than children with SpO_2_ ≥ 90%, but only IL-5 and G-CSF remained significant when adjusting for age in linear regressions. Children with crepitations had significantly higher concentrations of IL-1β (p = 0.032), IL-6 (p = 0.042), IL8 (p = 0.001), G-CSF (p < 0.001) and TNF-a (p = 0.003), but the significance of IL-1β and IL-6 disappeared when adjusting for age. Children with wheezing had significantly lower IP-10 (p = 0.001), which remained significantly associated also after adjusting for age (Table 3 and Table B in S1 File).

**Table 3:** Cytokine concentrations and differences in sub-populations based on CRP, SpO_2_, creptiations and wheezing.

Cytokine	CRP (mg/L)		SpO_2_		Crepitations		Wheezing	
	≥ 40	< 40	<90%	≥90%	Yes	No	Yes	No
IL-1β					1.5	1.4		
					**p = 0.032**			
IL-1ra			590	284.1				
			**p = 0.01**					
IL-2			31.5	21.8				
			**p = 0.036**					
IL-5			8.4	2				
			**p = 0.009**					
IL-6	90.8	37.9	83.4	40.7	47.4	39.3		
	**p < 0.001**		**p = 0.012**		**p = 0.042**			
IL-7			12	6.7				
			**p = 0.045**					
IL-8					27.3	22.5		
					**p = 0.001**			
IL-9			51	35				
			**p = 0.019**					
G-CSF	131.6	51.4	163.1	69.8	91.4	59.9		
	**p < 0.001**		**p = 0.018**		**p < 0.001**			
TNF-α			30.2	17.4	20.5	16.6		
			**p = 0.018**		**p = 0.003**			
IP-10	1308.6	1790.9					1336.5	1936.6
	**p = 0.007**						**p = 0.001**	
MCP1	143.8	195.4						
	**p = 0.026**							
MIP-1β	89	102.1						
	**p = 0.019**							

Table 3 shows the cytokines with significant differences in various sub-groups. Medians are given in absolute concentrations (pg/ml) and all p-values are based on the Mann–Whitney U- test.

There were no significant differences for children who were wasted (-2 Z-scores weight for height/length). For children who were stunted (-2 Z-scores height/length for age) there were significantly lower concentrations of IL-5 (p = 0.002), IL-9 (p = 0.033), IL-15 (p = 0.008) and TNF- α (p = 0.049) (Table 4).

**Table 4:** Cytokine concentrations and differences in sub-populations based on nutritional status, breastfeeding and exposure to indoor smoking.

Cytokine	Stunted		Breastfeeding		Exposure to	indoor
						smoking
	Yes	No	Yes	No	Yes	No
IL-4					1.9	2.2
					**p = 0.01**	
IL-5	1.2	2.1	2	4.6		
	**p = 0.002**		**p = 0.023**			
IL-9	31.9	36.8				
	**p = 0.033**					
IL-13					5.7	6.7
					**p = 0.041**	
IL-15	1.9	7				
	**p = 0.008**					
b-FGF			101.9	86.3	98.1	106.4
			**p = 0.025**		**p = 0.008**	
G-CSF					60.3	76.7
					**p = 0.007**	
GM-CSF			491.4	158.7	424.3	543.5
			**p = 0.009**		**p < 0.001**	
TNF-α	16.2	18.2			** **	** **
	**p = 0.049**				** **	** **
MIP-1α	** **	** **			4.4	4.8
	** **	** **			**p <**	**0.001 **
PDGF-BB	** **	** **			1204.1	1453.7
	** **	** **			**p = 0.022**	
VEGF	** **	** **			82.7	100.8
	** **	** **			**p = 0.005**	

Table 4 shows the cytokines with significant differences in various sub-groups. Medians are given in absolute concentrations (pg/ml) and all p-values are based on the Mann–Whitney U- test.

None of these associations remained significant after adjusting for age in linear regressions (Table C in S1 File). Children who were breastfed (94.9%) had significantly lower IL-5 (p = 0.023), and significantly higher b-FGF (p = 0.025) and GM-CSF (p = 0.009) (Table 4), IL-5 and G-CSF were still significantly associated after adjusting for age. Children exposed to indoor smoking had significantly lower plasma-concentration of IL-4 (p = 0.010), IL-13 (p = 0.041), basic-FGF (p = 0.008), G-CSF (p = 0.007), GM-CSF (p < 0.001), MIP-1α (p < 0.001), PDGF-BB (p = 0.022) and VEGF (p = 0.005) (Table 4). All these associations, except for PDGF-BB, remained significant after adjusting for age (Table C in S1 File).

The associations between the various viruses and cytokine concentrations are shown in Table D and Table E in S1 File. Albeit there were some statistically significant associations but none of the viruses yielded a clear, distinct cytokine response.

**Fig 1:**
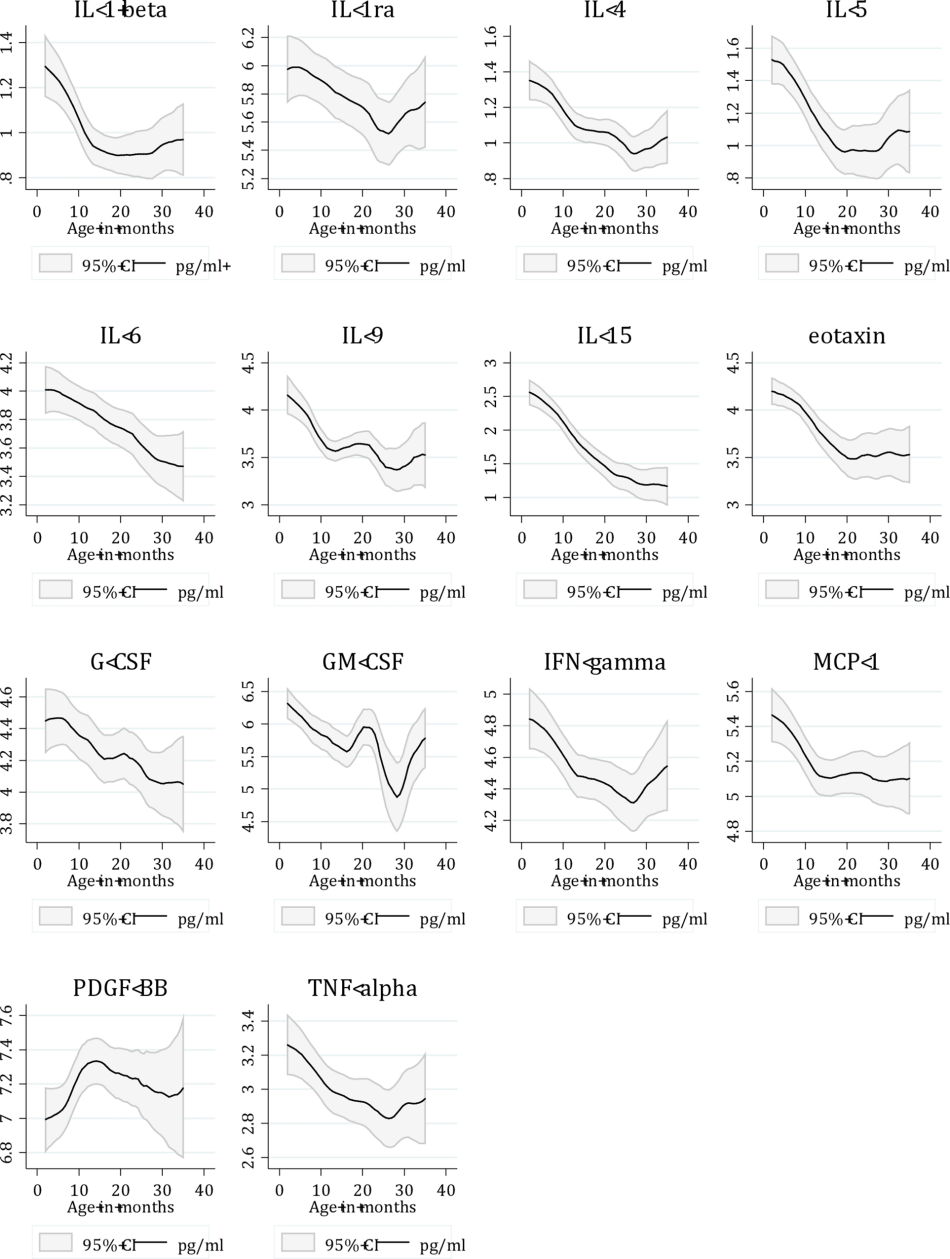
Cytokine concentrations in relation to age in a sample of Nepalese children with WHO defined severe or non-severe pneumonia. Cytokine concentrations are shown as natural log-values. Only cytokines with significant differences are shown.

For several of the cytokines, the concentrations were related to age (Fig 1).

The concentrations of IL-1β (p < 0.001), IL-1ra (p = 0.046), IL-4 (p < 0.001), IL-5 (p < 0.001), IL-6 (p < 0.001), IL-9 (p < 0.001), IL-15 (p < 0.001), eotaxin (p < 0.001), G-CSF (p = 0.013), GM-CSF (p = 0.003), IFN-γ (p = 0.005), MCP-1 (p < 0.001) and TNF-α (p = 0.010) were significantly negatively associated with age in linear regressions both when dichotomizing the variable in infants < 12 months and children 12–35 months and when adjusting for severity, and all remained significant associated, except IL-1ra, when keeping age as a continuous variable. (Table F in S1 File).

## Discussion

We measured the plasma concentrations of a wide range of cytokines in children with WHO-defined severe and non-severe CAP to describe the inflammatory response in childhood pneumonia and to measure the association between cytokines, severity and detection of viruses.

When comparing the groups of children with non-severe and severe pneumonia, we found that several cytokines were positively associated with severity, with generally higher concentrations of both predominantly pro-inflammatory and anti-inflammatory cytokines in the group of severe CAP compared to the children with non-severe CAP. This is in consistency with other studies on inflammation in pneumonia [6, 13]. Of the cytokines with predominantly pro-inflammatory effects investigated in our study, there were significantly higher concentrations of IL-1, IL-6 and TNF- in the plasma from children with severe pneumonia compared to the children with non-severe pneumonia. Of the cytokines with ability to down-regulate the production of pro-inflammatory cytokines, there were significantly higher concentrations of IL-4 in the patients with severe pneumonia compared to the group with non-severe pneumonia, but there were no significant differences in IL-10, which also is regarded as a predominantly anti-inflammatory cytokine. In previous reports, particularly the pro-inflammatory cytokine IL-6 and the anti-inflammatory cytokine IL-10 have been positively correlated to different markers of severity such as mental confusion, hypotension, pleural effusion, and bacteremia [14–16]. In one study of 38 adults with severe CAP, day 2 concentrations of IL-6 > 87 pg/ml and IL-10 >14.7 pg/ml also predicted mortality with a positive predictive value of 83% for each of these cytokines and a negative predictive value of respectively 94% and 91% [17]. In a study of the systemic and local cytokine profile including non-severe and severe adult CAP patients, Paats et al. concluded that the pro-inflammatory cytokines IL-6 and IFN-γ, together with the anti-inflammatory cytokine IL-10, are possible biomarkers of severity in CAP, which in their study was reflected by elevated systemic levels [13]. A similar study in 55 children with CAP published by Michelow et al. in 2007 also found a similar association between IL-6 and disease severity, but the children in this study were in general older and other markers of severity were used (white blood cell-band- forms, elevated procalcitonin and unequivocal consolidation) [5]. In our study IL-6 correlated positively with clinical severity, CRP ≥ 40 mg/L and SpO_2_ < 90% in the univariate analyses.

We also found that children with severe pneumonia had elevated concentrations of G-CSF, GM-CSF and IL-8. In response to LPS, TNF-α and IL-1 alveolar macrophages release G-CSF. G-CSF regulates the neutrophil production under healthy conditions and under infections [18]. It promotes survival of neutrophils [19] which may prolong the inflammatory response, but G-CSF is also a stimulator of the release of IL-1ra, soluble TNF-receptor (s-TNF-R) and IL-10 with anti-inflammatory properties [20]. G-CSF is raised in blood more rapidly than CRP in infections, and previous reports both in children and adults have confirmed that elevated levels are associated with bacterial agents and severity of illness [21–23]. There was significantly higher concentration of G-CSF in the severe group compared to the non-severe group, which could be an expression of bacterial infections or co-infections in these cases but we do not have information on bacterial etiology. We also found a significantly higher concentration of GM-CSF in plasma from children with severe disease. Research on animal models has shown that elevated expression of GM-CSF enhances innate immunity, and has the potential to reduce morbidity and mortality due to influenza virus [24]. The chemokine IL-8 was also significantly elevated in the group with severe pneumonia. Eotaxin, which is shown to be a potent chemo attractant inducing eosinophils, basophils, neutrophils and macrophages [25], was also significantly elevated in the group with severe pneumonia. Additionally, we found significantly higher concentrations of IL-9, IL-15 and basic fibroblast growth factor (b-FGF) in the group of children with severe CAP compared to the children with non-severe CAP. IL-9 is a pleiotropic interleukin, earlier classified as a Th2-interleukin and has the potential to activate several immune cells, including mast cells in lung and gut tissue [26]. Experiments in murine models have shown that overexpression of IL-9 in the lung is associated to excessive inflammatory response [27]. IL-15 is important for anti-viral function especially through the induction of NK-cell proliferation [28, 29]. Elevated concentrations of basic fibroblast growth factor have been demonstrated during lower airway infections [30] and may be associated with airway remodelling [31].

The variables CRP, SpO_2_, presence of crepitations and wheezing are closely connected to the severity of pneumonia and we wanted to look at sub-populations based on these variables. The variables were positively correlated with several cytokines in the univariate analyses, but many of these associations disappeared after adjusting for age. G-CSF was the cytokine that most consistently was associated with these variables, also after adjusting for age. IL-6 was also associated in the univariate analysis for CRP, SpO_2_ and presence of crepitations, but this association was substantially attenuated and not longer significant when adjusting for age. All cytokine concentrations associated to stunting disappeared when adjusting for age, reflecting that these associations were confounded by age. Breastfeeding status was to a limited extent associated with differences in cytokine concentrations, but the disproportionate number of breastfed versus non-breastfed in the study make this result difficult to interpret. Exposure to indoor smoking was negatively correlated with concentration of the anti-inflammatory cytokines IL-4 and IL-13, all the growth factors and MIP-1α. A decreased production of several pro- and anti-inflammatory cytokines and chemokines is previously demonstrated in healthy adult smokers [32].

We observed a positive correlation between the concentrations of some of the cytokines with certain viruses detected in nasopharyngeal aspirate. In a study in adults by Menèndez et al. it was shown that the level of IL-10 was higher and the level of TNF-α lower in pneumonia caused by influenza virus compared to bacterial causes [33]. In another study in paediatric patients by Kim et al the concentrations IL-6 and IP -10 were found to be higher in patients with influenza A/H1N1 and pneumonia than those without H1N1-infection [6]. Berdal et al reported similar findings in adult patients [34]. Influenza A has been shown to induce both IL-8 and GM-CSF [35]. We found relatively few associations of cytokine concentrations with different viruses, but most of the associations for Influenza A and RSV remained significant after adjusting for age, and may reflect the degree of inflammation associated to these two viruses. The lack of significant associations observed may reflect that virus etiology was less important for the inflammatory response in these children or that our PCR assay lacked sensitivity for certain viruses. Notably, except RSV, the viruses were detected in low numbers, which may also affect our results [11].

Several of the cytokines were negatively correlated with age. The production of cytokines such as IL-6 and TNF- increases to adult levels within the first three years of life, and IFN-γ- and IL-12-production may remain low until teenage [3, 36]. Out of the total patients, 201 were < 12 months of age, and these children may be likely to have a less mature cytokine response, which may explain the relatively low concentrations of IFN-γ and TNF-. The cytokine concentrations were in general higher in the infants than in the older children however, thus the opposite of what was expected, and the associations remained significant also after adjusting for severity.

Two general challenges in the interpretation of cytokine concentrations are the lack of established normal ranges, and the limited knowledge about age differences. We did not have plasma from healthy controls in this study, which makes the results more difficult to interpret. One study from 2013 by Kleiner et al. compared concentrations of 48 cytokines in 72 healthy individuals aged 1–86 years in order to describe cytokine concentrations by age [37]. Compared to the children aged 1–6 years in this study, the hallmark pro-inflammatory cytokines such as IL-1, IL-2 and IL-6 and anti-inflammatory cytokines such as IL-10 were generally higher in our patients. On the other hand, concentrations of the pro-inflammatory cytokines such as IL-12 (p70), TNF-α and IFN-γ and the concentrations of the immune modulating cytokines with anti-inflammatory effects such as IL-4 and IL-13 were overall lower in our children than in this sample of healthy individuals. Because of the limited number of children and differences in mean age and ethnicity, this comparison should be done with caution, however. There are also some other limitations in this study. The sample consists of primarily non-severe cases, which may affect the precision of our estimates when comparing the concentration with severe cases. We do not have information on bacterial etiology, which probably contributes to disease severity, alone or in combination with other respiratory pathogens in several of the cases. Additional measurements of cytokine concentrations during the first few days could have given a more complete picture of the dynamics of the concentrations of the different cytokines. Moreover, the plasma specimens were stored for 10 years before analysis, and the plasma was stored on heparinized-tubes, while the preferred tubes for cytokine analyses are EDTA or citrate plasma. Interference in the analysis could have lead to the lower concentrations that were measured for some of the cytokines. However, the levels of cytokines found in our study are comparable with other studies. Strengths of this study are the relatively large sample of patients and the wide range of cytokines analysed.

To conclude, our main findings in this study were that the plasma concentrations of several inflammatory mediators with both pro- and anti-inflammatory effects, chemotactic and growth stimulatory effects were generally higher among children with severe CAP than in children with non-severe CAP, which reflect the degree of immune activation in these groups. The cytokines that most consistently reflected severity in the univariate analyses in this study were G-CSF and IL-6, which supports previous reports of studies both in children and adults. Unexpectedly, the cytokine responses were negatively associated with age. There was also little variation in cytokine response according to viral etiology. The results indicate that immunological markers may provide supplemental information to clinical findings and assessments. The results may also indicate that the inflammatory profile correlate stronger to the clinical signs of severity and age than to the certain viruses detected in this study. Studies with identification of a broader panel of pathogens could elucidate this further.

## Supporting Information

S1 Fig (TIFF)

S1 File Table A, Table B, Table C, Table D, Table E, Table F. (DOCX)

S2 File Excel file with raw data. (XLS)

S3 File Variable codes. (DOCX)
