# Supplementary material for: Cytokine Concentrations in Plasma from Children with Severe and Non-Severe Community Acquired Pneumonia
Source: PLoS One. 2015 Sep 25;10(9):e0138978. doi: 10.1371/journal.pone.0138978 (PMC4583304; doi:10.1371/journal.pone.0138978)
Supplement: S1 File — (DOCX) [file pone.0138978.s002.docx]

**SUPPLEMENTAL INFORMATION**

**Table A. Regression model for associations of cytokine concentrations and severity, adjusted for age.**

| **Cytokine** | **Association** | **to severity** | **(unadj.)** | **Association** | **to severity** | **(adj. for age)** |
| --- | --- | --- | --- | --- | --- | --- |
|  | **Coeff.** | **p-value** | **95% C.I.** | **Coeff.** | **p-value** | **95% C.I.** |
| IL-1β | 0.3 | **0.005** | 0.09 – 0.51 | 0.21 | 0.052 | -0.002 – 0.43 |
| IL-1ra | 0.34 | 0.095 | -0.06 – 0.75 | 0.26 | 0.214 | -0.15 – 0.67 |
| IL-2 | 0.14 | 0.376 | -0.17 – 0.44 | 0.1 | 0.513 | -0.21 – 0.42 |
| IL-4 | 0.28 | **0.001** | 0.11 – 0.45 | 0.19 | **0.025** | 0.02 – 0.36 |
| IL-5 | 0.37 | **0.014** | 0.07 – 0.66 | 0.23 | 0.129 | -0.07 – 0.53 |
| IL-6 | 0.36 | **0.022** | 0.05 – 0.66 | 0.24 | 0.13 | -0.07 – 0.55 |
| IL-7 | 0.28 | 0.091 | -0.04 – 0.60 | 0.23 | 0.178 | -0.10 – 0.56 |
| IL-8 | 0.39 | **<0.001** | 0.19 – 0.59 | 0.4 | **<0.001** | 0.20 – 0.60 |
| IL-9 | 0.52 | **<0.001** | 0.25 – 0.79 | 0.38 | **0.006** | 0.11 – 0.65 |
| IL-10 | 0.13 | 0.45 | -0.20 – 0.45 | 0.13 | 0.445 | -0.21 – 0.47 |
| IL-12p70 | 0.06 | 0.733 | -0.29 – 0.41 | -0.01 | 0.938 | -0.37 – 0.35 |
| IL-13 | 0.27 | 0.057 | -0.01 – 0.54 | 0.22 | 0.122 | -0.06 – 0.50 |
| IL-15 | 0.76 | **<0.001** | 0.38 – 1.14 | 0.39 | **0.03** | 0.04 – 0.75 |
| IL-17a | 0.4 | 0.103 | -0.04 – 0.46 | 0.22 | 0.104 | -0.04 – 0.48 |
| eotaxin | 0.4 | **0.013** | 0.09 – 0.71 | 0.21 | 0.186 | -0.10 – 0.52 |
| b-FGF | 0.26 | **0.005** | 0.08 – 0.44 | 0.26 | **0.005** | 0.08 – 0.44 |
| G-CSF | 0.7 | **<0.001** | 0.37 – 1.0 | 0.62 | **<0.001** | 0.29 – 0.96 |
| GM-CSF | 0.46 | **0.027** | 0.05 – 0.86 | 0.27 | 0.194 | -0.14 – 0.68 |
| *IFN-γ | 0.36 | **0.025** | 0.05 – 0.68 | 0.26 | 0.109 | -0.06 – 0.59 |
| IP-10 | 0.04 | 0.804 | -0.29 – 0.37 | 0.1 | 0.544 | -0.23 – 0.44 |
| MCP-1 | 0.15 | 0.23 | -0.10 – 0.40 | 0.06 | 0.654 | -0.20 – 0.31 |
| MIP-1α | 0.06 | 0.313 | -0.06 – 0.19 | 0.04 | 0.494 | -0.08 – 0.17 |
| MIP-1β | 0.01 | 0.917 | -0.18 – 0.20 | 0.01 | 0.953 | -0.19 – 0.21 |
| PDGF-BB | -0.15 | 0.296 | -0.44 – 0.13 | -0.11 | 0.45 | .-0.41 – 0.18 |
| RANTES | -0.34 | 0.321 | -1.01 – 0.33 | -0.6 | 0.086 | -1.28 – 0.08 |
| TNF-α | 0.43 | **0.005** | 0.13 – 0.73 | 0.35 | **0.027** | 0.04 – 0.65 |
| VEGF | 0.05 | 0.674 | -0.18 – 0.27 | 0.03 | 0.819 | -0.20 – 0.26 |

* Not significant in Mann-Whitney-U test

**Table B.** **Regression table for cytokine concentrations and associations to CRP ≥ 40, CRP as a continuous variable and associations to SpO_2_ < 90% and SpO_2_ as continuous variable.**

| **Cytokine** | **Association** | **to variable** | **(unadjusted)** | **Association** | **to variable** | **(adjusted for age)** |
| --- | --- | --- | --- | --- | --- | --- |
|  | **Coeff.** | **p-value** | **95% C.I.** | **Coeff.** | **p-value** | **95% C.I.** |
| **Association** | **to CRP** ≥ **40** | (CRP ≥ 40 | yes=1, no=0) |  |  |  |
| IL-6 | 0.72 | **<0.001** | 0.46 – 0.97 | 0.66 | **<0.001** | 0.41 – 0.92 |
| G-CSF | 0.66 | **<0.001** | 0.37 – 0.94 | 0.61 | **<0.001** | 0.33 – 0.89 |
| IP-10 | -0.33 | **0.021** | -0.61 – -0.05 | -0.31 | **0.032** | -0.59 – -0.03 |
| MCP-1 | -0.25 | **0.02** | -0.46 – -0.04 | -0.3 | **0.005** | -0.51 – -0.09 |
| *MIP-1β | -0.13 | 0.125 | -0.30 – 0.04 | -0.31 | 0.116 | -0.30 – 0.03 |
| **Association** | **to CRP** | (continous) |  |  |  |  |
| IL-6 | 0.01 | **<0.001** | 0.008 – 0.014 | 0.01 | **<0.001** | 0.008 – 0.014 |
| G-CSF | 0.01 | **<0.001** | 0.007 – 0.014 | 0.01 | **<0.001** | 0.006 – 0.014 |
| MCP-1 | -0.003 | **0.041** | -0.006 – 0.0001 | -0.003 | **0.017** | -0.006 – 0.0006 |
| MIP-1β | -0.002 | **0.049** | -0.004 – 0.000008 | -0.002 | **0.046** | -0.004 – 0.00004 |
| **Association** | **to SpO_2_ <** | **90%** | (SpO_2_ < 90 yes=1, | no=0) |  |  |
| *IL-1ra | 0.63 | 0.109 | -0.14 – 1.39 | 0.57 | 0.14 | -0.19 – 1.33 |
| *IL-2 | 0.36 | 0.221 | -0.22 – 0.94 | 0.34 | 0.249 | -0.24 – 0.92 |
| IL-5 | 0.81 | **0.005** | 0.25 – 1.37 | 0.73 | **0.009** | 0.18 – 1.27 |
| IL-6 | 0.6 | **0.042** | 0.02 – 1.18 | 0.53 | 0.069 | -0.04 – 1.10 |
| *IL-7 | 0.42 | 0.182 | -0.20 – 1.03 | 0.38 | 0.22 | -0.12 – 1.00 |
| IL-9 | 0.58 | **0.029** | 0.06 – 1.09 | 0.48 | 0.058 | -0.02 – 0.99 |
| G-CSF | 0.83 | **0.011** | 0.19 – 1.46 | 0.77 | **0.017** | 0.14 – 1.40 |
| *TNF-α | 0.51 | 0.081 | -0.06 – 1.08 | 0.45 | 0.117 | -0.11 – 1.02 |
| **Association** | **to SpO_2_** | (continuous) |  |  |  |  |
| *IL-1ra | 0 | 0.73 | -0.003 – 0.002 | 0 | 0.71 | -0.003 – 0.002 |
| *IL-2 | -0.00. | 0.367 | -0.001 – 0.003 | 0 | 0.375 | -0.001 – 0.003 |
| IL-5 | 0 | 0.217 | -0.003 – 0.001 | 0 | 0.187 | -0.003 – 0.001 |
| IL-6 | 0 | 0.61 | -0.003 – 0.002 | 0 | 0.572 | -0.003 – 0.001 |
| *IL-7 | 0 | 0.691 | -0.003 – 0.002 | 0 | 0.675 | -0.003 – 0.002 |
| IL-9 | 0 | 0.708 | -0.002 – 0.002 | 0 | 0.649 | -0.002 – 0.001 |
| G-CSF | 0.83 | 0.011 | 0.003 – 0.002 | 0 | 0.81 | -0.003 – 0.002 |
| *TNF-α | 0.51 | 0.081 | -0.002 – 0.002 | 0 | 0.954 | -0.002 – 0.002 |

**Table C**. **Regression table for cytokine concentrations associated to crepitations, wheezing,**

**stunting, current breastfeeding, indoor smoking, adjusted for age.**

| **Cytokine** | **Association** | **to variable** | **(unadj.)** | **Association** | **to variable** | **(adj. for age)** |
| --- | --- | --- | --- | --- | --- | --- |
|  | **Coeff.** | **p-value** | **95% C.I.** | **Coeff.** | **p-value** | **95% C.I.** |
| **Association to crepitation** |  | (Crepitations | yes=1, no=2) |  |  |  |
| *IL-1β | -0.06 | 0.412 | -0.20 – 0.08 | -0.07 | 0.298 | -0.21 – 0.06 |
| *IL-6 | -0.18 | 0.079 | -0.37 – 0.02 | -0.2 | **0.049** | -0.39 – -0.00 |
| IL-8 | -0.21 | **0.001** | -0.34 – -0.08 | -0.21 | **0.001** | -0.34 – -0.08 |
| G-CSF | -0.31 | **0.006** | -0.52 – -0.09 | -0.32 | **0.003** | -0.53 – -0.11 |
| TNF-α | -0.27 | **0.007** | -0.46 – -0.07 | -0.28 | **0.004** | -0.47 – -0.09 |
| **Association to wheezing** |  | (Wheezing | yes=1, no=0) |  |  |  |
| *G-CSF | -0.12 | 0.11 | -0.27 – -0.03 | -0.11 | 0.157 | -0.26 – 0.04 |
| IP-10 | 0.23 | **0.002** | 0.08 – 0.37 | 0.22 | **0.003** | 0.07 – 0.36 |
| MCP-1 | 0.13 | **0.021** | 0.02 – 0.24 | 0.14 | **0.01** | 0.03 – 0.25 |
| **Association to stunting** |  | (Stunted | yes=1, no=1) |  |  |  |
| IL-5 | -0.34 | **0.002** | -0.55 – -0.13 | -0.19 | 0.086 | -0.42 – 0.03 |
| *IL-9 | -0.17 | 0.084 | -0.37 – 0.02 | 0.01 | 0.92 | -0.19 – 0.21 |
| IL-15 | -0.4 | **0.005** | -0.67 – -0.12 | 0.04 | 0.753 | -0.22 – 0.31 |
| *TNF-α | -0.21 | 0.062 | -0.42 – 0.01 | 0.11 | 0.355 | -0.34 – 0.12 |
| **Association to br.feeding** |  | (Current | breastfeeding | yes=1, no=0) |  |  |
| IL-5 | 0.45 | **0.033** | 0.04 – 0.86 | 0.68 | **0.001** | 0.27 – 1.08 |
| *b-FGF | -0.2 | 0.113 | -0.45 – 0.05 | -0.2 | 0.13 | -0.45 –0.06 |
| GM-CSF | -1.44 | **<0.001** | -1.99 – -0.89 | -1.24 | **<0.001** | -1.79 – -0.68 |
| **Association to smoking** |  | (Exposed to | smoking | yes=1, no=0) |  |  |
| IL-4 | 0.14 | **0.008** | 0.04 – 0.24 | 0.14 | **0.007** | 0.04 – 0.24 |
| IL-13 | 0.19 | **0.022** | 0.03 – 0.36 | 0.19 | **0.023** | 0.03 – 0.36 |
| b-FGF | 0.13 | **0.022** | 0.02 – 0.23 | 0.13 | **0.023** | 0.02 – 0.23 |
| G-CSF | 0.29 | **0.005** | 0.09 – 0.49 | 0.29 | **0.005** | 0.09 – 0.49 |
| GM-CSF | 0.43 | **0.001** | 0.19 – 0.67 | 0.43 | **<0.001** | 0.19 – 0.66 |
| MIP-1α | 0.17 | **<0.001** | 0.10 – 0.24 | 0.17 | **<0.001** | 0.10 – 0.24 |
| *PDGF-BB | 0.15 | 0.087 | -0.02 – 0.33 | 0.15 | 0.083 | -0.02 – 0.33 |
| VEGF | 0.18 | **0.009** | 0.04 – 0.31 | 0.18 | **0.01** | 0.04 – 0.31 |

*Significant in non-parametric test only

**Table D. Cytokine concentrations and associations to isolated virus in NPA.**

| **Cytokine** | **PIV** | **1** | **PIV** | **2** | **PIV** | **3** | **Influensa** | **A** | **Hmpv** |  | **RSV** |  |
| --- | --- | --- | --- | --- | --- | --- | --- | --- | --- | --- | --- | --- |
|  | **Yes** | **No** | **Yes** | **No** | **Yes** | **No** | **Yes** | **No** | **Yes** | **No** | **Yes** | **No** |
|  | **n= 20** | **n= 364** | **n= 5** | **n= 379** | **n= 32** | **n= 352** | **n= 23** | **n= 361** | **n= 16** | **n= 368** | **n= 60** | **n= 324** |
| IL-1β |  |  |  |  |  |  |  |  | 1.4 | 0.9 |  |  |
|  |  |  |  |  |  |  |  |  | **p =** | **0.003** |  |  |
| IL-4 | 3 | 2 |  |  |  |  |  |  | 1.2 | 2 |  |  |
|  | **p =** | **0.045** |  |  |  |  |  |  | **p =** | **0.039** |  |  |
| IL-7 |  |  |  |  |  |  | 3.3 | 6.6 |  |  |  |  |
|  |  |  |  |  |  |  | **p =** | **0.026** |  |  |  |  |
| IL-8 |  |  | 32.2 | 23.1 | 25.7 | 6.6 |  |  |  |  | 29.5 | 22.5 |
|  |  |  | **p =** | **0.036** | **p =** | **0.041** |  |  |  |  | **p <** | **0.001** |
| IL-9 |  |  |  |  |  |  |  |  | 48.6 | 36.5 |  |  |
|  |  |  |  |  |  |  |  |  | **p =** | **0.049** |  |  |
| IL-13 |  |  |  |  | 8.2 | 6.2 |  |  |  |  |  |  |
|  |  |  |  |  | **p =** | **0.009** |  |  |  |  |  |  |
| IL-15 |  |  |  |  |  |  |  |  |  |  | 9.3 | 4.6 |
|  |  |  |  |  |  |  |  |  |  |  | **p =** | **0.041** |
| IFN-γ | 140.1 | 83.2 |  |  | 113.4 | 81.5 |  |  |  |  |  |  |
|  | **p =** | **0.044** |  |  | **p =** | **0.009** |  |  |  |  |  |  |
| IP-10 |  |  |  |  |  |  | 2961.8 | 1577.4 |  |  |  |  |
|  |  |  |  |  |  |  | **p =** | **0.008** |  |  |  |  |
| MIP-1β |  |  |  |  |  |  |  |  |  |  | 84.9 | 97.3 |
|  |  |  |  |  |  |  |  |  |  |  | **p =** | **0.002** |
| PDGF-BB |  |  |  |  |  |  |  |  |  |  | 1094.7 | 1443.6 |
|  |  |  |  |  |  |  |  |  |  |  | **p =** | **0.009** |

The table shows median values of cytokine concentration and differences between cytokine concentrations in plasma from children with detection of six certain viruses in NPA and those with no detection of the certain viruses. Only the significant differences are shown, and the group with influenza B is therefore omitted because there were no significant differences in this group. P-values are based on Mann-Whitney U-test and a value < 0.05 was considered significant.

**Table E. Regression table for cytokine concentration associated to virus detection in NPA.**

| **Cytokine** | **Unadjusted** |  |  | **Adjusted for** | **age** |  |
| --- | --- | --- | --- | --- | --- | --- |
|  | **Coeff.** | **p-value** | **95% C.I.** | **Coeff.** | **p-value** | **95% C.I.** |
| **Parainfluenza virus 1** |  |  |  |  |  |  |
| *IL-4 | 0.19 | 0.09 | -0.03 – 0.42 | 0.22 | 0.053 | -0.003 – 0.43 |
| *IFN-γ | 0.35 | 0.118 | -0.09 – 0.78 | 0.37 | 0.091 | -0.06 – 0.80 |
| **Parainfluenza virus 2** |  |  |  |  |  |  |
| *IL-8 | 0.44 | 0.099 | -0.08 – 0.96 | 0.43 | 0.106 | -0.09 – 0.95 |
| **Parainfluenza virus 3** |  |  |  |  |  |  |
| *IL-8 | 0.13 | 0.219 | -0.08 – 0.35 | 0.13 | 0.222 | -0.08 – 0.35 |
| *IL-13 | 0.24 | 0.212 | -0.06 – 0.54 | 0.24 | 0.122 | -0.06 – 0.53 |
| *IFN-γ | 0.35 | 0.052 | -0.003 – 0.69 | 0.34 | 0.052 | -0.002 – 0.69 |
| **Influenza virus A** |  |  |  |  |  |  |
| IL-7 | -0.53 | **0.017** | -0.96 – 0.09 | -0.56 | **0.011** | -1.00 – -0.13 |
| IP-10 | 0.56 | **0.006** | 0.16 – 0.95 | 0.59 | **0.004** | 0.19 – 0.99 |
| **Human metapneumovirus** |  |  |  |  |  |  |
| *IL-1β | -0.08 | 0.509 | -0.15 – 0.31 | -0.13 | 0.414 | -0.45 – 0.18 |
| *IL-4 | -0.09 | 0.329 | -0.09 – 0.27 | -0.12 | 0.332 | -0.36 – 0.12 |
| IL-9 | 0.61 | **0.002** | 0.23 – 0.99 | 0.62 | **0.001** | 0.25 – 0.99 |
| **Respiratory syncytial virus** |  |  |  |  |  |  |
| IL-8 | 0.28 | **0.001** | 0.12 – 0.44 | 0.27 | **0.001** | 0.11 – 0.43 |
| IL-15 | 0.41 | **0.02** | 0.07 – 0.75 | 0.28 | 0.075 | -0.03 – 0.59 |
| MIP-1β | -0.24 | **0.001** | -0.39 –-0.10 | -0.25 | **0.001** | -0.40 – -0.11 |
| PDGF-BB | -0.31 | **0.006** | -0.54 – -0.09 | -0.31 | **0.007** | -0.53 – -0.08 |

The regression table is based on the significant results from Mann-Whitney-U-test (Table 6). Regression coefficients are based on log-values of the cytokine concentrations. * Significant in non-parametric test only.

**Table F. Regression table of associations of cytokine concentrations and age, adjusted for severity.**

| **Cytokine** | **Association** | **to age <12** | **months** | **Association** | **to age < 12** | **months** |
| --- | --- | --- | --- | --- | --- | --- |
|  |  | **(unadj.)** |  | **(adj. for** | **severity)** |  |
|  | **Coeff.** | **p-value** | **95% C.I.** | **Coeff.** | **p-value** | **95% C.I.** |
| IL-1β | -0.31 | **<0.001** | -0.44 – -0.19 | -0.29 | **<0.001** | -0.41 – -0.16 |
| IL-1ra | -0.29 | **0.021** | -0.53 – -0.04 | -0.25 | **0.046** | -0.50 – 0.004 |
| IL-4 | -0.25 | **<0.001** | -0.35 – -0.15 | -0.23 | **<0.001** | -0.33 – -0.13 |
| IL-5 | -0.54 | **<0.001** | -0.71 – -0.37 | -0.52 | **<0.001** | -0.69 – -0.34 |
| IL-6 | -0.34 | **<0.001** | -0.52 – -0.15 | -0.3 | **0.001** | -0.49 – -0.12 |
| IL-9 | -0.42 | **<0.001** | -0.58 – -0.26 | -0.37 | **<0.001** | -0.53 – -0.21 |
| IL-15 | -0.99 | **<0.001** | -1.2 – -0.78 | -0.94 | **<0.001** | -1.15 – -0.72 |
| eotaxin | -0.57 | **<0.001** | -0.75 – -0.38 | -0.54 | **<0.001** | -0.73 – -0.35 |
| G-CSF | -0.34 | **0.001** | -0.54 – -0.14 | -0.26 | **0.013** | -0.46 – -0.05 |
| GM-CSF | -0.42 | **0.001** | -0.66 – -0.17 | -0.37 | **0.003** | -0.62 – -0.13 |
| IFN-γ | -0.32 | **0.001** | -0.51 – -0.12 | -0.28 | **0.005** | -0.48 – -0.09 |
| MCP-1 | -0.29 | **<0.001** | -0.44 – -0.14 | -0.28 | **<0.001** | -0.43 – -0.13 |
| PDGF-BB | 0.2 | **0.024** | 0.03 – 0.37 | 0.19 | **0.037** | 0.01 – 0.37 |
| TNF-α | -0.29 | **0.002** | -0.47 – -0.11 | -0.24 | **0.01** | -0.43 – -0.06 |
|  |  |  |  |  |  |  |
| **Cytokine** | **Association** | **to continous** | **age** | **Association** | **to continous** | **age** |
|  |  | **(unadj.)** |  | **(adj. for** | **severity)** |  |
|  | **Coeff.** | **p-value** | **95% C.I.** | **Coeff.** | **p-value** | **95% C.I.** |
| IL-1β | -0.02 | **<0.001** | -002 – -0.01 | -0.01 | **<0.001** | -0.02 – -0.01 |
| IL-1ra | -0.01 | **0.038** | -0.03 – -0.00 | -0.01 | 0.081 | -0.03 – 0.002 |
| IL-4 | -0.02 | **<0.001** | -0.02 – -0.01 | -0.01 | **<0.001** | -0.02 – -0.008 |
| IL-5 | -0.02 | **<0.001** | -0.03 – -0.01 | -0.02 | **<0.001** | -0.03 – -0.01 |
| IL-6 | -0.02 | **<0.001** | -0.03 – -0.01 | -0.02 | **0.001** | -0.03 – -0.01 |
| IL-9 | -0.03 | **<0.001** | -0.03 – -0.02 | -0.02 | **<0.001** | -0.03 – -0.01 |
| IL-15 | -0.06 | **<0.001** | -0.07 – -0.05 | -0.06 | **<0.001** | -0.07 – -0.05 |
| eotaxin | -0.03 | **<0.001** | -0.04 – -0.02 | -0.03 | **<0.001** | -0.04 – -0.02 |
| G-CSF | -0.02 | **0.003** | -0.03 – -0.01 | -0.01 | **0.037** | -0.02 – -0.0008 |
| GM-CSF | -0.03 | **<0.001** | -0.05 – -0.02 | -0.03 | **<0.001** | -0.04 – -0.01 |
| IFN-γ | -0.02 | **0.002** | -0.03 – -0.01 | -0.02 | **0.008** | -0.03 – -0.004 |
| MCP-1 | -0.02 | **0.001** | -0.02 – -0.01 | -0.01 | **0.001** | -0.02 – -0.006 |
| TNF-α | -0.02 | **0.004** | -0.03 – -0.01 | -0.01 | **0.019** | -0.02 – -0.002 |
