# Supplementary material for: Cytokine Concentrations in Plasma from Children with Severe and Non-Severe Community Acquired Pneumonia
Source: PLoS One. 2015 Sep 25;10(9):e0138978. doi: 10.1371/journal.pone.0138978 (PMC4583304; doi:10.1371/journal.pone.0138978)
Supplement: S3 File — (DOCX) [file pone.0138978.s004.docx]

Variable codes

znumolL1 = baseline zinc in umol/L

zlen = length for age z-score

zwei = weight for age z-score

zwfl = weight for length z-score

crp = crp

crp40 = crp40==1: crp ≥ 40, crp==0: crp < 40

crp80 = crp80==1: crp ≥ 80, crp==0: crp < 80

c_5 = age

c_7 = Is the child unable to drink/breast feed? Yes ==1, No ==0

c_8 = Does the child vomit out all he/she eats? Yes ==1, No ==0

c_9 = Is there any history og convulsions? Yes ==1, No ==0

c_10 = Is the child lethargic/ unconscious? Yes ==1, No ==0

c_11 = Duration og cough (in days)

c_12 = Duration og difficult breathing (in days)

c_14 = Respiratory rate (after nebulizer/salbutamol) Yes ==1, No==0

c_16 = Lower chest indrawings Yes==1, No ==0

c_19wheezepos = Wheezing Yes ==1, No ==0

c_22 = Crepitations Yes ==1, No ==2

d_10 = Duration og fever (in days)

d_11 = Temperature (in Fahrenheit)

d_21 = Weight (in kg)

d_22 = Height/length (in cm)

b_4 = Sex: Male ==1, Female ==2

b_32 = Does the child still breast feed? Yes ==1, No ==2

a_12 = No. of family members

a_15 = Family ownership of agricultural land Yes ==1, No ==2

a_19 = Indoor smoking? Yes ==1, No ==2

p1pos = Positive for parainfluenza 1 Yes ==1, No ==0

p2pos = Positive for parainfluenza 2 Yes ==1, No ==0

p3pos = Positive for parainfluenza 3 Yes ==1, No ==0

iapos = Positive for influenza A Yes ==1, No ==0

ibpos = Positive for influenza B Yes ==1, No ==0

rsvpos = Positive for respiratory syncytial virus Yes ==1, No ==0

hmpvpos = Positive for human metapneumovirus Yes ==1, No ==0

sp02 = oxygen saturation (in %)

hypox90 = oxygen saturation < 90% Yes ==1, No ==0

severe = severe pneumonia Yes ==1, No ==0

h_duration = time till recovery (in days)

il1betapgml1 = baseline concentration of IL-1beta in pg/ml

etc.

The variable codes show how the different variables in the excel file are coded.
